# Maternal, fetal, neonatal and breastmilk flecainide concentration during maternal therapy and lactation: a case report

**DOI:** 10.1186/s13006-023-00559-z

**Published:** 2023-04-14

**Authors:** Johanna A. van der Zande, Jérôme M.J. Cornette, Jolien W. Roos-Hesselink, Robert B. Flint

**Affiliations:** 1 Department of Cardiology, Erasmus MC, University Medical Center Rotterdam, Rotterdam, the Netherlands; 2 Department of Obstetrics and Gynecology, Erasmus MC – Sophia Children’s Hospital, University Medical Center Rotterdam, Rotterdam, The Netherlands; 3 Department of Hospital Pharmacy, Erasmus MC, University Medical Center Rotterdam, PO Box: 2040, Rotterdam, 3000 CA The Netherlands; 4 Department of Neonatal and Pediatric Intensive Care, Division of Neonatology, Erasmus MC – Sophia Children’s Hospital, University Medical Center Rotterdam, Rotterdam, The Netherlands

**Keywords:** Breastfeeding, Flecainide, Pregnancy, Maternal medication use, Neonatal blood sampling

## Abstract

**Background:**

Mothers requiring the antiarrhythmic agent flecainide are often advised not to breastfeed, because of the lack of data concercing neonatal effects and flecainide plasma concentrations following maternal exposure as well as via lactation. This is the first report on combined maternal, fetal, neonatal and breastmilk flecainide concentrations in a breastfed infant of a mother requiring flecainide treatment.

**Case presentation:**

A 35-year old Gravida 2 Para 1, known with ventricular arrhythmia, was referred to our tertiary center at 35 + 4 weeks of gestation. Because of an increase of ventricular ectopy, oral metoprolol 11.9 milligrams once daily was switched to oral flecainide 87.3 milligrams twice daily. Weekly collected maternal flecainide plasma trough concentrations fell within the therapeutic range of 0.2 to 1.0 mg/L and no further clinically significant arrhythmias occurred during the study period. A healthy son was born at 39 weeks of gestation and had a normal electrocardiogram. The fetal to maternal flecainide ratio was 0.72 and at three different timepoints, the flecainide concentration was higher in breastmilk than in maternal plasma. The relative infant dose received via breastmilk compared to maternal dose was 5.6%. Neonatal plasma concentrations were not detectable, despite the flecainide passage into breastmilk. All electrocardiograms to assess the neonatal antiarrhytmic effect were normal.

**Conclusions:**

Our results assume that flecainide can be prescribed safely to lactating mothers. Quantification of drug concentrations in neonatal blood in addition to measurements in maternal and fetal blood, and breastmilk, are helpful to evaluate the effects and safety of maternal medication use during pregnancy and lactation.

## Case report

### Background

The majority of pregnant women use at least one medication at any time in pregnancy and will face a decision about taking medicines during nursing [1]. Because of lack of information on possible teratogenic and adverse effects on fetus and neonates, mothers are often advised not to breastfeed or avoid taking medications if possible. Flecainide, a potent class Ic antiarrhythmic agent, is used to prevent and treat maternal and fetal arrhythmias. Its lipophilicity and moderate protein binding contribute to high placental passage to fetus and passage into breastmilk [2]. Data are scarce concerning neonatal effects and flecainide plasma concentrations following maternal exposure as well as via lactation [3, 4]. Therefore, mothers requiring flecainide are often advised not to breastfeed, despite the general perception of an acceptable safety profile. In cases like this, at our institution, we introduced weekly capillary blood samples in the newborn to investigate the flecainide concentration in neonatal blood as well as the concentration in breastmilk. Our report describes a woman needing flecainide for maternal ventricular arrhythmia during pregnancy and after delivery with the flecainide plasma concentrations in the mother, fetus, breastmilk as well as in the infant.

### Case presentation

A 35-year old woman, Gravida 2 Para 1, was referred in January 2021 to our tertiary center at 35 + 4 weeks of gestation. She was known to have ventricular arrhythmia previously responding well to flecainide treatment. Due to fetal concerns, flecainide was switched to oral metoprolol 11.9 milligrams once daily (split tablet of metoprololsuccinaat 23.75 mg, slow release, Sandoz, Switzerland) in a local hospital at 9 weeks of gestation. At 35 + 4 weeks of gestation, metoprolol was stopped because of an increase of ventricular ectopy and oral flecainide 87.3 milligrams twice daily (flecainideacetaat 100 mg, Sandoz, Switzerland) was restarted. Weekly collected maternal flecainide plasma trough concentrations fell within the therapeutic range of 0.2 to 1.0 mg/L (Table 1) and no further clinically significant arrhythmias occurred during the study period. Flecainide plasma and breastmilk concentrations were quantified using liquid chromatography with mass spectrometric detection (LC-MS/MS, Thermo TSQ Vantage, Thermo Fisher Scientific, Waltham, US). The flecainide range of linearity was 0.05-10.0 mg/L. The fetus showed normal 1/1 atrioventricular conduction on ultrasound with a fetal heart rate of 120–140 bpm. At 39 + 0 weeks of gestation, a son with a birthweight of 2860gr (8th percentile) was born after spontaneous vaginal delivery with a good start (Apgar 5 min score of 10). The fetal to maternal flecainide ratio was 0.72, based on the umbilical cord fetal concentration of 0.26 mg/L and 0.36 mg/L in maternal blood, although the maternal plasma sample was collected 3 days before delivery. The infant was monitored on the maternity ward for 24 h and showed a heart rate of 125 bpm and had a normal electrocardiogram (ECG). Maternal to breastmilk and neonatal transfer of flecainide were monitored by weekly maternal plasma, breastmilk, and neonatal plasma samples and are presented in Table 1, including the time after dose. The infant was exclusively breastfed. At three different timepoints, the flecainide concentration in breastmilk was 2.3-fold, 2.7-fold and 3.3-fold higher than in maternal plasma. Despite the flecainide passage into breastmilk, neonatal plasma concentrations were not detectable (lower limit of quantification of 0.05 mg/L).

**Table 1:** Flecainide concentration and time after dose in maternal blood, breastmilk and infant blood

Date and Time	Time after dose (Hours)^#^	Flecainide concentration (mg/L)			Milk / plasma
		Maternal blood	Breastmilk	Infant blood	
January 26, 2021 (8:39AM)	0.7	0.25			
February 1, 2021 (9:13AM)	1.2	0.29			
February 9, 2021 (9:19AM)	1.3	0.36			
February 12, 2021 (3:44PM) **Delivery**	7.7			0.26*	
February 13, 2021 (1:30PM)	5.5		0.55		
February 16, 2021 (12:00PM)	4.0		0.94		
February 19, 2021 (1:30PM)	5.5		0.77		
February 22, 2021 (1:22PM)	5.4			< 0.05	
February 22, 2021 (1:58PM)	6.0	0.41	0.94		2.3
February 25, 2021 (1:00PM)	5.0		0.94		
March 1, 2021 (9:40AM)	1.7	0.32	0.87		2.7
March 1, 2021 (10:23AM)	2.4			< 0.05	
March 8, 2021 (9:07AM)	1.1	0.30	0.99		3.3
March 8, 2021 (9:30AM)	1.5			< 0.05	
March 15, 2021 (9:23AM)	1.4	0.33			
April 12, 2021 (10:30AM)	2.5		0.61		
April 12, 2021 (10:50AM)	2.8			< 0.05	
April 12, 2021 (11:54AM)	3.9	0.36			
May 25, 2021 (10:44AM)	2.7	0.53			
August 16, 2021 (9:48AM)	1.8			< 0.05	
August 16, 2021 (11:25AM)	3.4	0.67			

^#^ Compared to daily flecainide administration at 8:00AM and 8:00PM.

* Blood collected from the umbilical cord after delivery

The worst-case absolute infant dose (AID) was 0.15 mg/kg/day, based on the highest measured flecainide concentration of 0.99 mg/L in breastmilk and a consumed volume of 150 ml/kg/day. The relative infant dose received via breastmilk compared to maternal dose (RID) of 2.7 mg/kg/day (= 175 mg/64 kg bodyweight) was 5.6% [5]. The relative infant dose received via breastmilk compared to the infant therapeutic flecainide dosage of 2 mg/kg/day was 7.5%. Neonatal antiarrhythmic effect was assessed by weekly neonatal ECG, which were all normal.

## Discussion and conclusions

This is the first report on combined maternal, fetal, breastmilk and neonatal flecainide concentrations in a breastfed infant of a mother requiring flecainide treatment. The low RID, AID, undetectable flecainide concentration in neonatal blood, and absent cardiac symptoms suggest that flecainide can safely be used during lactation, despite flecainide passage into breastmilk.

The RID is often used to evaluate the effect of maternal medication use on the breastfed infant, and an RID below 10% is considered safe [5]. Two previous studies estimated the RID instead of flecainide quantification in neonatal blood and found proportions of 3.6% and 4.5% [3, 4]. Our RID was 5.6%, which is quite comparable with the previous studies. In addition, the AID was 0.15 mg/kg/day which is much lower than the therapeutic dose in neonates of 2–8 mg/kg/day [6]. However, it has to be considered that the RID is only a safety threshold for risk assessment and not a toxicity threshold. The drug’s pharmacological action and the infants’ metabolic capacities are not taken into account. Therefore, combined drug concentrations in maternal plasma, fetal plasma (collected from umbilical blood), breastmilk and infant plasma are required to adequately evaluate perinatal drug exposure in relation to its effects and safety. As breastmilk constitution, intake and infant’s intestinal drug absorption evolve over time, repeated measurements are recommended. Neonatal blood sampling can be performed with simple heel prick, or using scavenged blood samples. A previous focus series on drug exposure in newborns of mothers using medication during pregnancy and lacation also described the added value of measurements of drug concentrations in the newborn in evaluating the safety of breastfeeding [7]. However, in our knowledge, this method is hardly ever applied.

In conclusion, based on our and two previous published reports, flecainide can be prescribed safely to lactating mothers as neonatal plasma concentrations are undetectable despite passage into breastmilk. For essential maternal drugs of which safety during breastfeeding has not yet been established, simultaneous measurement of drug concentrations in maternal blood, fetal blood, breastmilk and infant blood are a valuable aid to acertain neonatal safety and enhance maternal reassurance and comfort with breastfeeding. Systematic collection and pooling of these data will provide valuable evidence to establish the safety of perinatal drug use and allow breastfeeding in the future.

## Data Availability

The dataset used and/or analysed during the current study is available from the corresponding author on reasonable request.

## Abbreviations

AID: absolute infant dose
ECG: electrocardiogram
RID: relative infant dose

## References

[1] Mitchell AA (2011). Medication use during pregnancy, with particular focus on prescription drugs: 1976–2008. Am J Obstet Gynecol.

[2] Palmer CM, Norris MC (1990). Placental transfer of flecainide. Am J Dis Child.

[3] McQuinn RL (1990). Flecainide excretion in human breast milk. Clin Pharmacol Ther.

[4] Wagner X (1990). Coadministration of flecainide acetate and sotalol during pregnancy: lack of teratogenic effects, passage across the placenta, and excretion in human breast milk. Am Heart J.

[5] Rowe H, Baker T, Hale TW (2013). Maternal medication, drug use, and breastfeeding. Pediatr Clin North Am.

[6] *Nederlands Kenniscentrum voor Farmacotherapie bij Kinderen (NKFK) (c2008). Dutch Paediatric Formulary - Flecainide-acetate. Retrieved: 31 August* 2022.; Available from: https://www.kinderformularium.nl/geneesmiddel/399/flecainide-acetaat.

[7] Rentsch KM (2020). Drug exposure in newborns: effect of selected drugs prescribed to mothers during pregnancy and lactation. Ther Drug Monit.

